# Case Based Learning in Psychiatry: Use of Interactive Presentation Software and Fictional Narrative

**DOI:** 10.1192/bjo.2024.311

**Published:** 2024-08-01

**Authors:** Gareth McGuigan, Scott Barr, Megan Robertson, Syeda Ghouri, Ayemyat Doris

**Affiliations:** 1 NHS Forth Valley, Larbert, United Kingdom; 2 University of Glasgow, Glasgow, United Kingdom; 3 NHS GG&C, Glasgow, United Kingdom; 4 NHS A&A, Kilmarnock, United Kingdom

## Abstract

**Aims:**

Undergraduate Psychiatry placements often struggle to provide the bedside teaching familiar to students from other specialties. Efforts to reproduce this experience in tutorials can be impaired by lack of interactivity, high student-to-teacher ratio, and use of mostly didactic pedagogy. Psychiatry trainees have provided weekly tutorials in ‘Clinical Skills' to University of Glasgow students on Psychiatric placement for several years. Unfortunately, these tutorials suffered from poor attendance, poor engagement, and difficulty recruiting facilitators. We created an afternoon of teaching structured around three presentations of a fictional patient in a narrative fashion aimed at solving these issues and providing excellent experience for students.

**Methods:**

Together with Glasgow University tutors, we selected Learning Objectives that would benefit from additional formal teaching. We then created a fictionalised patient narrative incorporating presentations of self-harm, delirium and postnatal depression. Teaching materials were created using mentimeter.com to allow for maximal engagement and interactivity. The content included brief summary slides, groupwork, Word-Clouds, anonymous quizzes, and simulated clinical encounters/roleplay. Custom illustrated vignettes accompanied each scenario to increase verisimilitude. The day is delivered by three Psychiatry trainees to up to forty students in their penultimate week of placement. Feedback is gathered digitally and anonymously on the day.

**Results:**

77/80 students invited attended. 71 (92%) completed feedback: 100% ranked the day positively - either *“very helpful”* (85.9%) or “*somewhat helpful”* (14.1%). Students advised it was *“extremely useful”* preparation for both clinical placements (73.2%) and exams (88.7%). All attendees provided free-text remarks; quotes include *“One of the best teaching days I've been to”* and *“Best teaching of the block”*. 84.5% felt *“very involved”* in the day and the word *“interactive”* was used 30 times in freetext. When asked on what could be improved, the most common response was *“another session”* (34%).

**Conclusion:**

Recruitment to Psychiatry relies on positive experiences during placement. Retention of Psychiatrists relies on providing rewarding and varied working experiences. Our hope is that successful events like this support both aims. The creative use of narrative, illustrated vignettes, roleplay and interactive questions afforded excellent engagement and enjoyable experiences for student and facilitator, as reflected in the feedback.

Going forward, we plan to refine this case and develop another. We are seeking review and design input from patient representatives and EDI experts. Comparison of students' exam outcomes and feedback from the replaced tutorials is also planned. Use of this format across other specialties is also being pursued.

